# Heterotrophic Bioleaching of Vanadium from Low-Grade Stone Coal by Aerobic Microbial Consortium

**DOI:** 10.3390/ijerph192013375

**Published:** 2022-10-17

**Authors:** Han Zhang, Jiaxin Shi, Cuibai Chen, Meng Yang, Jianping Lu, Baogang Zhang

**Affiliations:** MOE Key Laboratory of Groundwater Circulation and Environmental Evolution, School of Water Resources and Environment, China University of Geosciences (Beijing), Beijing 100083, China

**Keywords:** vanadium, bioleaching, stone coal, heterotrophic microbial consortium

## Abstract

Bioleaching is a viable method that assists in increasing the vanadium output in an economical and environmentally friendly manner. Most bioleaching is conducted by pure cultures under autotrophic conditions, which frequently require strong acidity and produce acid wastewater. However, little is known about heterotrophic bioleaching of vanadium by mixed culture. This study investigated the bioleaching of vanadium from low-grade stone coal by heterotrophic microbial consortium. According to the results, vanadium was efficiently extracted by the employed culture, with the vanadium recovery percentage in the biosystem being 7.24 times greater than that in the control group without inoculum. The average vanadium leaching concentration reached 680.7 μg/L in the first three cycles. The kinetic equation indicated that the main leaching process of vanadium was modulated by a diffusion process. Scanning electron microscopy revealed traces of bacterial erosion with fluffy structures on the surface of the treated stone coal. X-ray photoelectron spectroscopy confirmed the reduction of the vanadium content in the stone coal after leaching. Analysis of high-throughput 16S rRNA gene sequencing revealed that the metal-oxidizing bacteria, *Acidovorax* and *Delftia*, and the heterotrophic-metal-resistant *Pseudomonas*, were significantly enriched in the bioleaching system. Our findings advance the understanding of bioleaching by aerobic heterotrophic microbial consortium and offer a promising technique for vanadium extraction from low-grade stone coals.

## 1. Introduction

Vanadium exists ubiquitously in the Earth’s crust and is widely employed in modern industries [1,2]. Nowadays, over 85% of vanadium production is used for manufacturing carbon steel, stainless steel and ferrovanadium for its ability to alter physical properties of hardness, tensile strength and fatigue resistance [3,4]. It is also used as an oxidation catalyst and cracking agent in the chemical industry. Aerospace industry also utilizes vanadium extensively for the manufacture of titanium–aluminum alloys [5]. Additionally, a vanadium redox battery is another potential application of vanadium [6,7]. Thus, the demand for vanadium is growing in modern industry. China, South Africa and Russia are currently the world’s leading vanadium-producing countries, with vanadium harvested mainly from ores, concentrates and vanadiferous slag [8]. In addition to vanadium, titanomagnetite, a vanadium-bearing stone coal, is another source for vanadium with huge reserves. The available vanadium reserves in stone coal account for more than 87% of the total vanadium reserves in China, which is about 6.7 times that of vanadium titanomagnetite [9,10]. Therefore, vanadium extraction from stone coal is increasingly focused [11].

Stone coal occurs naturally in carbonaceous shale with a relatively lower calorific value of 4.18 MJ/kg and is mainly formed prior to the start of middle Devonian Epoch [12]. Both inorganic and organic components are found in stone coal, with the carbon content mostly ranging from 10% to 20%. For stone coals, vanadium mainly exists in the crystal lattice of the aluminosilicate minerals. Some also complex with organic compounds as isomorphism and/or adsorb on the surface of clay and pyrite [12]. However, the mass fraction of vanadium in stone coal is only 0.13% to 1.2%. As high as 60% of stone coal contains a vanadium content less than the cut-off grade level of 0.5% [13]. Various processes have been reported for vanadium extraction from stone coal with a vanadium content greater than the cut-off grade, such as roasting, acid leaching, calcination and deposition [14]. However, most low-grade stone coal is not economically viable as vanadium-containing feedstocks due to high mining costs, low efficiency and the operation complexity of conventional chemical processes [15,16]. Furthermore, conventional methods also cause serious environmental challenges due to the release of poisonous gases, wastewater and solid residues [17]. To solve these problems, it is essential to develop new vanadium extracting methods with high efficiency, low cost and environmental soundness to recover vanadium from low-grade stone coal [18].

Bioleaching is introduced as an environment-friendly method requiring less complexity and cost in operation [19,20], and can be an alternative to chemical extraction. In fact, vanadium has been recovered from industrial wastes such as stone coal [21], spent refinery catalysts [22], vanadium-bearing shale and slag fly ash through bioleaching process [23,24]. Chemolithotrophic bacteria, such as the *Acidithiobacillus* species, are well known for their use in metal bioleaching (e.g., copper, gold, and uranium) from ore deposits and mine tailings [25]. However, bioleaching by chemolithotrophic bacteria usually requires a strong acid environment, which leads to the production of massive amounts of acidic wastewater as secondary pollution. Compared with chemolithotrophic bacteria, heterotrophic microorganisms utilize a wider array of metabolic pathways, using organics as carbon sources for the synthesis of organic acids. The bioprocess coupling to the acidity generated by organic metabolites promotes the bioleaching process [26,27]. Moreover, bioleaching by heterotrophic microorganisms can break the metal–oxygen bond and release the metal by directly consuming the organic components in ores. The viability of this method makes bioleaching of great importance for stone-coal mining [28]. In addition, the vanadium extraction tailings produced by the bioleaching of stone coal can be used to prepare improved materials, such as ceramsite, which are widely used as fillers in the construction of constructed wetlands [29].

Our research is the first study to investigate the feasibility of vanadium bioleaching from low-grade stone coal using aerobic heterotrophic microbial consortium as inoculum. Surface morphology and mineral composition of the stone coal before and after bioleaching were examined. The microbial community composition was explored in conjunction with microbial interaction patterns. Collectively, these findings developed a method for bioleaching under mild conditions, providing an environment-friendly strategy for vanadium recovery from low-grade stone coal.

## 2. Materials and Methods

### 2.1. Experimental Materials and the Inoculum

Stone coal was obtained from Huitong mine, Huaihua City of Hunan Province, China, which is famous for its vanadium resources [30]. Before the experiment, the stone coal was mechanically crushed and sieved to less than 74 μm (200-mesh). Mixed aerobic sludge was collected from the Beijing Gaobeidian Wastewater Treatment Plant as inoculum.

### 2.2. Bioreactor Setting-Up and Experimental Operations

Six plexiglass columns (17 cm in height, 3.8 cm in diameter) were employed and connected with an air inflator, providing a constant airflow rate (6.0 mL/min) (Figure S1, Supporting Information). The top of the column was covered with a 4.5 cm diameter lid to reduce water evaporation. All columns were operated with 2 g stone coal as reactant. Six plexiglass columns were divided equally into three groups. The first group (Bioreactors) was inoculated with 5 mL aerobiotic sludge as well as 95 mL medium (2.05 g/L acetate, 0.07 g/L KH_2_PO_4_, 0.31 g/L NH_4_Cl), while the second group (Control 1) was composed of 5 mL aerobiotic sludge and 95 mL deionized water addition. In addition, Control 2 constituted another 2 reactors filled with 100 mL deionized water but without inoculum as abiotic controls. All chemicals used in this study were analytical grade reagents and all aqueous solutions were prepared using deionized water.

The original inoculum was cultivated steadily in Bioreactors and Control 1 groups by refreshing the stone coal and aqueous solution every 72 h. At the end of each cycle, the supernatant was filtered through 0.22 μm microporous membrane to retain the microorganisms and placed the microbe-laden membrane back into the columns for further domestication without significant biomass loss. After 30 d incubation, Bioreactors and Control 1 achieved a steady state at room temperature (22 ± 2 °C). Bioleaching experiments were initiated in plexiglass columns and lasted for six consecutive cycle (18 d) studies in three groups, recording variations in vanadium concentrations at the column effluent to evaluate the performance of the bioleaching process. In addition, COD concentration was determined at the end of each cycle in Bioreactor. Furthermore, the concentrations of other elements (e.g., Ca and Mg) were also measured during the first cycle. All experiments were carried out in triplicate at an ambient temperature (22 ± 2 °C).

### 2.3. Analytical Methods

All aqueous samples were passed through 0.22 μm filters before analysis. Total vanadium and other metals in the aqueous solution were determined by ICP-MS (Thermo Fisher X series, Waltham, MA, USA). COD concentration was measured by fast airtight-catalytic-decomposition method [31]. Stone coal was crushed by the sealed sampling crusher (BULL, China) to attain fine-powder samples before physical characterization. The surface morphology of stone coal was examined by scanning electron microscope (SEM) (Quanta, FEI Co., Hillsboro, OR, USA). Components of stone coal were determined by X-ray fluorescence (XRF, F7000, Hitachi, Japan) and X-ray photoelectron spectroscopy (XPS, Axis Ultra, Kratos Analytical Ltd., Manchester, UK), using the fine powder. The stone-coal structures were analyzed by X-ray diffraction (XRD) using Cu-Kα (λ = 1.5405 A) as radiation source, with an operating voltage of 40 kV and 200 mA (Rigaku-D/MAX-PC 2500, Rigaku, Japan).

### 2.4. Microbiological Analysis

At the end of experiments, the microorganisms attached to the surfaces of the stone coal in Bioreactor and Control 1 were collected. Then, DNA extraction and 16S rRNA gene sequencing were performed on the collected microbial samples together with inoculum. Total genomic DNA was extracted from samples using Fast DNA SPIN Kit for Soil (MP Biomedicals, Santa Ana, CA, USA) according to the manufacturer’s instructions. DNA was amplified by PCR (GeneAmp^®^ 9700, ABI, Waltham, MA, USA) using primers 338F (ACTCCTACGGGAGGCAGCAG) and 806R (GGACTACHVGGGTWTCTAAT). Purified PCR products were quantified using a QuantiFluorTM-ST microfluorometer (Promega, Madison, WI, USA). After being purified and quantified, a mixture of amplicons was used for high-throughput 16S rRNA gene sequencing on MiSeq (Illumina, San Diego, CA, USA). The resulting data were processed, and microbial communities were analyzed, as previously described [32,33].

## 3. Results and Discussion

### 3.1. Characteristics of the Stone Coal

**Figure 1:**
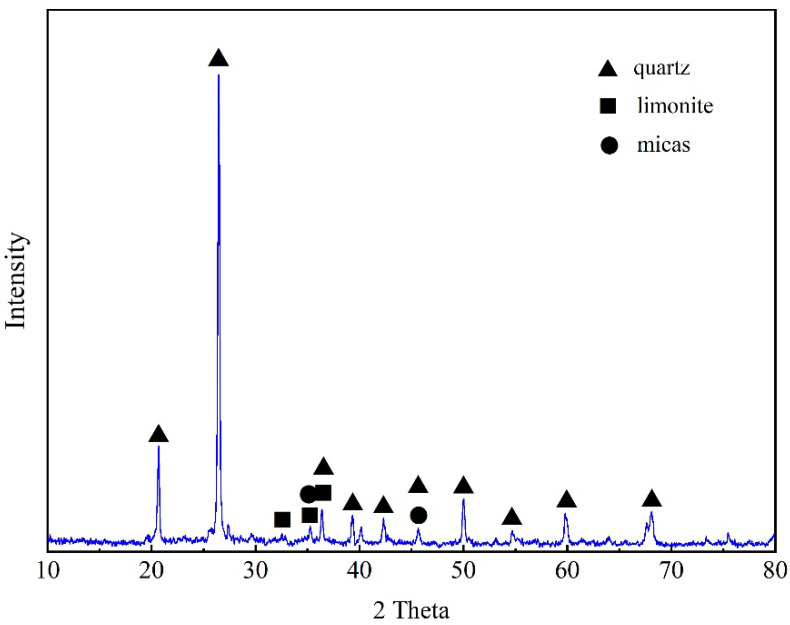
XRD characterization of stone coal.

The main chemical constituents of stone coal were analyzed by XRF (Table S1, Supporting Information). Si was the most abundant element detected in stone coal (32.56%), while Al was the highest among metal (3.37%), followed by Fe (2.95%). Vanadium accounted for 0.23%, indicating that the stone coal used in this study was a typical low-grade-vanadium-containing stone coal ore. Other metals were also detected in the stone coal, such as Ca (1.36%), Ti (0.82%), K (0.98%) and Mg (0.74%). XRD analysis was used to characterize the mineral composition of the stone coal, which existed mainly in a crystalline state composed of quartz, limonite and illite (Figure 1). Vanadium in stone coal existed in various forms, with a dependence on their ore of origin.

### 3.2. Bioleaching Performance

**Figure 2:**
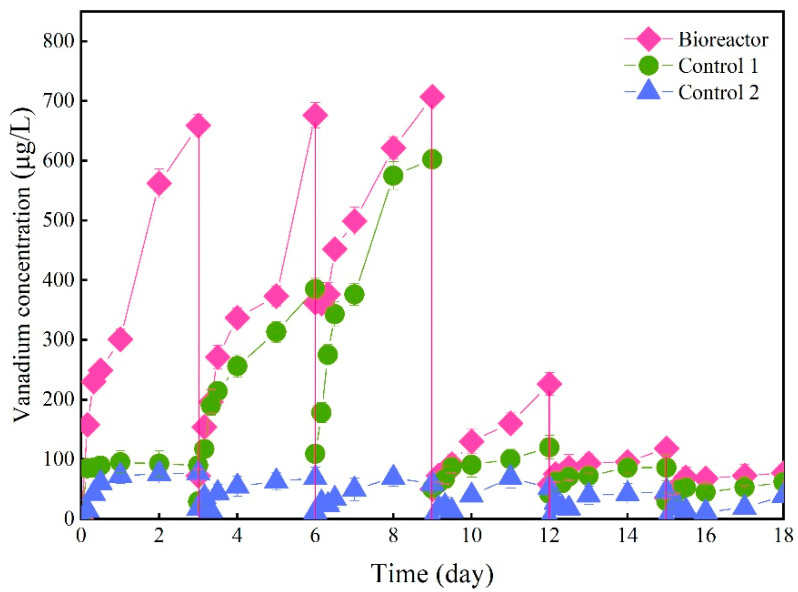
Variations of leached vanadium concentrations during six consecutive operating cycles.

The variations of bioleaching vanadium concentrations in different reactors were measured along six consecutive cycles (Figure 2). For Bioreactor, the vanadium concentration gradually increased with the leaching process in each cycle, with a higher concentration in Bioreactor than in Control 1 and Control 2. The vanadium leaching concentrations reached 659 μg/L, 676 μg/L and 707 μg/L, respectively, in the Bioreactor during the first three cycles. Some studies reported that heterotrophic microorganisms synthesize organic acids (citric, oxalic, gluconic etc.) as metabolic by-products during their cellular metabolism. The acidity generated by the produced organic metabolites and metal-chelating properties of organic compounds were responsible for the solubilization of metal from minerals [34,35]. After the third cycle, the leaching concentration of vanadium rapidly dropped in Bioreactor, which demonstrated the depletion of vanadium in stone coal. In addition, the vanadium concentration increased significantly in Control 1 after the first cycle, with the highest vanadium concentration reaching 602 μg/L in the third cycle. A possible explanation is that without the addition of a carbon source, the bacteria consumed the organics in the stone coal to sustain their bacterial growth, thus disrupting the stone-coal structure and significantly promoting vanadium leaching. Comparably, the leaching concentration of vanadium in each cycle of Control 2 was relatively low, with the highest concentration being up to 76.6 μg/L. The recovery of vanadium in Bioreactor, Control 1 and Control 2 were 5.43%, 2.96% and 0.75%, respectively. Similar results of low recovery efficiency were also obtained in the experiment of vanadium bioleaching from basalt [36]. Under the condition of a sufficient carbon source, the recovery of vanadium in Bioreactor was increased by 1.83 times and 7.24 times compared to Control 1 and Control 2, respectively, further demonstrating the important roles of bacteria in the bioleaching process.

Meanwhile, the variation of COD concentration in the Bioreactor was measured (Figure S2, Supporting Information), with a uniform decreasing trend from 1600 mg/L to about 100 mg/L during each cycle (72 h). The result indicated the utilization of organic carbon by heterotrophic microorganisms to maintain their own metabolic growth and participate in the leaching process. The results of the changes in the concentrations of other elements and pH during leaching process in Bioreactor are displayed in Figure S3, Supporting Information. The leaching concentrations of Ca and Mg followed a similar trend, wherein higher concentrations were obtained in the earlier cycles. Metabolites, such as organic acids produced by bacterial metabolism, lowered the system pH, facilitating the release of metal ions from stone coal. As the reaction proceeded, the bacteria depleted the organic acids and exopolysaccharides enriched in the system, which led to the recovery of pH, resulting in the formation of Ca- and Mg-rich sedimentary phases. The leaching process of Si overall showed an increasing trend. The main reason was that the microbially induced acid hydrolysis and the complexation of the extracellular polymer destroyed the structure of the aluminum silicate salt in the stone coal, resulting in the release of silicon [37].

### 3.3. Vanadium Leaching Kinetics

The bioleaching that occurred in the solid phase was controlled by the diffusion mass transfer of the generated ions through the solid–liquid boundary and the metal deposits. The process was generally characterized by both diffusion control and chemical-reaction control [38]. During the bioleaching, the reactants successively diffused through the fluid film surrounding the solid matrix while a chemical reaction was taking place. For metal leaching controlled by diffusion, the shrinking-core-model theory can be applied [39]:

(1) $$kt=1-\frac{2}{3}X-{\left(1-X\right)}^{\left(\frac{2}{3}\right)}$$

where *k* was the rate constant, *t* was the reaction time and *X* was the metal recovery efficiency. The chemical-reaction-controlled leaching process can be expressed using the following equation:

(2) $$kt=1-{\left(1-X\right)}^{\left(\frac{1}{3}\right)}$$

**Figure 3:**
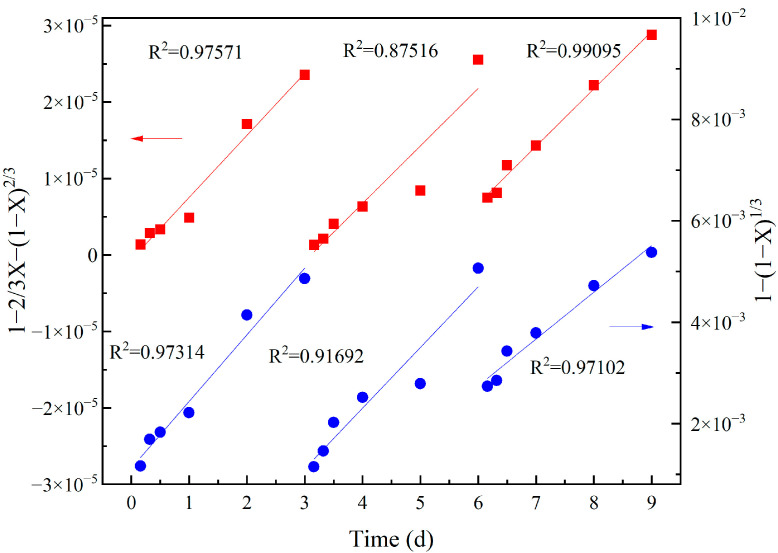
Kinetic model of vanadium leaching controlled by diffusion and chemical reaction in Bioreactor. Left axis provides measurement label of the diffusion model (red) and right axis corresponds to the chemical-reaction model (blue), as indicated by arrow.

The kinetic equations of diffusion- and chemical-reaction control were calculated for the Bioreactor during the first three cycles (Figure 3). According to the R^2^ value, the diffusion-controlled reaction was rather linear, indicating that the main leaching process of vanadium in Bioreactor was governed by the diffusion process. Meanwhile, the chemical-reaction control also exhibited a good fitting, implying the important role of chemical reaction to govern the vanadium leaching process. Similar results were also found in a study on the vanadium bioleaching from power-plant residual ash using organic acids produced by *Aspergillus niger* [38]. For Control 1 and Control 2, the diffusion control exerted more influence on the bioleaching process based on higher R^2^ (Figure S4, Supporting Information).

### 3.4. Morphological Characterization

**Figure 4:**
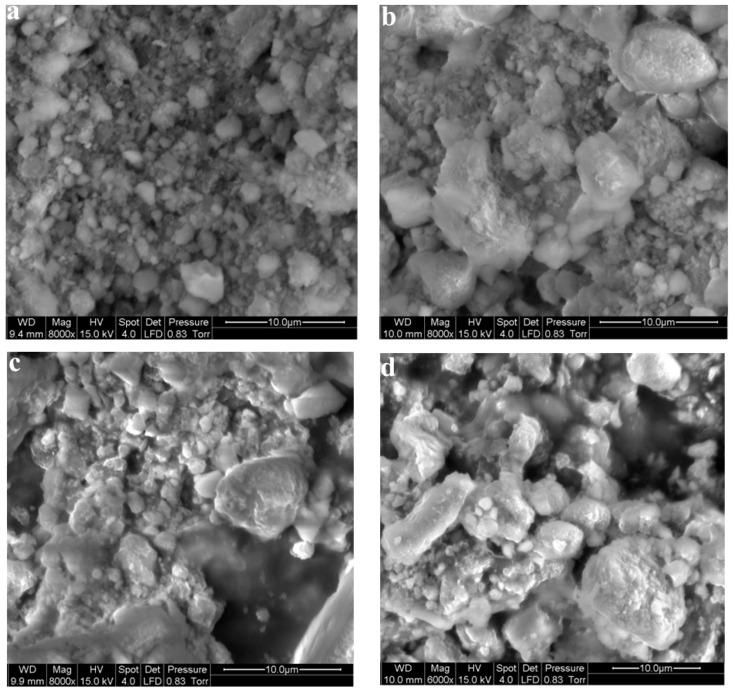
SEM images of solid stone coal. (**a**) Initial stone coal sample; (**b**) stone coal in Bioreactor after six cycle operation; (**c**) stone coal in Control 1 after six cycle operation; (**d**) stone coal in Control 2 after six cycle operation.

SEM analysis revealed the surface morphology of the stone coal before and after bioleaching (Figure 4). Compared with the raw ore, the surface of solid minerals treated by bacteria showed a fluffy structure with traces of microbial erosion. Irregular flocs appeared in the Bioreactor, indicating that bacteria played a key role in the bioleaching process. In Control 1, bacteria consumed the carbon in the stone coal to maintain bacterial metabolism in the absence of an added carbon source, exhibiting obvious signs of bacterial erosion. In Control 2, an insignificant amount of corrosion pits was also observed due to the leaching of small amounts of vanadium.

**Figure 5:**
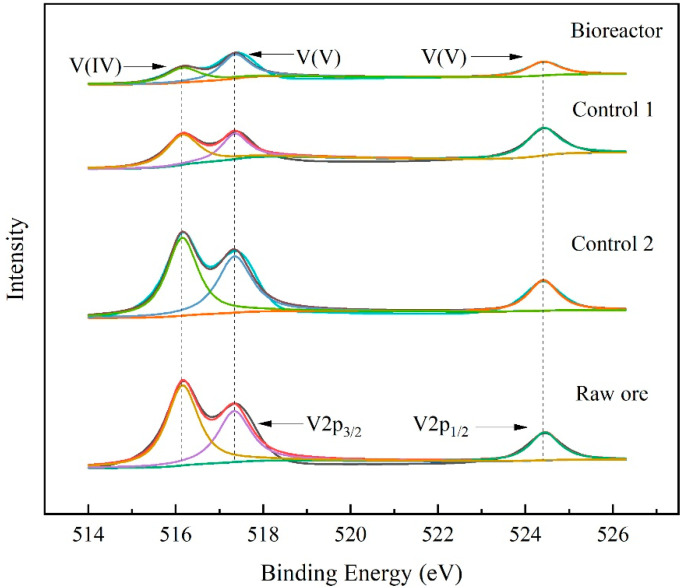
XPS spectra of the V 2P regions in the stone coal before and after leaching.

The stone coal surface before and after leaching was investigated by XPS (Figure 5). In general, the binding energy of V2p_3/2_ and V2p_1/2_ electrons was used to reflect the valence state of vanadium [40]. The electron-binding energies of V2p_3/2_ and V2p_1/2_ corresponding to V(V) were located at 517.42 eV and 524.44 eV, respectively, while the sub-band situated at 516.17 eV was identified to be V(IV) [41,42]. There is no special symmetrical peak in each spectrum, indicating that the peaks were formed by the superposition of different valence states of the vanadium element, and that the vanadium in the stone coal mainly existed in the form of V(IV) and V(V). According to the peak height, the contents of V(IV) and V(V) in Bioreactor and Control 1 were lower than that of the raw mineral ore, suggesting that a fraction of vanadium was already leached from stone coal and transferred into the solution. Weaker spectrum peaks of V(V) were obtained in Bioreactor than that in Control 1, suggesting a better leaching effect for Bioreactor, wherein heterotrophic microorganisms use an adequate carbon source to synthesize organic acids for vanadium bioleaching.

### 3.5. Microbial Community Characteristics

The microbial community structure changed significantly due to variations in environmental conditions. For alpha diversity, the Chao1 index, Ace index and Shannon index were reduced in Bioreactor and Control (Table S2, Supporting Information), indicating that the addition of stone coal reduced the richness and diversity of the microbial community, inhibiting the microorganisms as a result of metal leaching.

**Figure 6:**
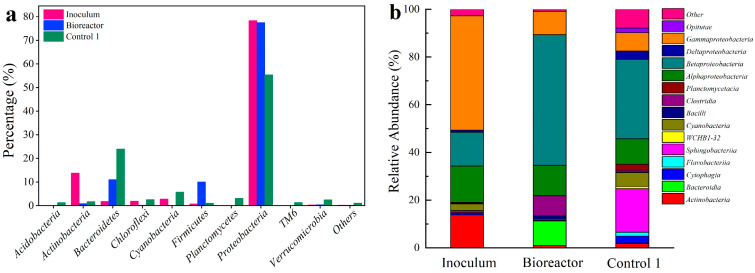

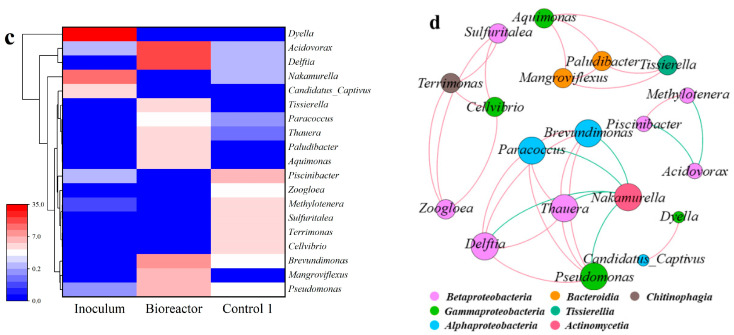
Microbial community composition and interactions. (**a**) The relative abundance of microorganisms at phylum level; (**b**) the relative abundance of microorganisms at class level; (**c**) heatmap of functional microorganisms at genus level; (**d**) co-occurrence network of functional microorganisms; the node represents genus. The size of the node is proportional to the degree of connectivity. Node colors indicate different classes. Positive and negative correlations between nodes were connected by red and green lines, respectively.

Microbial composition at the phylum level are shown in Figure 6a. The bacterial community changed significantly in the Bioreactor compared to the inoculum. The relative abundance of *Actinobacteria*, *Chloroflexi* and *Cyanobacteria* were almost undetectable, while *Bacteroidetes* (11.02%) and *Firmicutes* (10.03%) increased significantly in the Bioreactor. Additionally, *Acidobacteria* (1.35%), *Bacteroidetes* (24.01%) and *Planctomycetes* (3.09%) were enriched in Control 1. This result showed that microbial communities evolved in order to adapt to altered conditions during the bioleaching. The relative abundance of microbial community at the class level are shown in Figure 6b. *Gammaproteobacteria*, *Alphaproteobacteria* and *Betaproteobacteria* were more abundant in Bioreactor and Control 1. Among them, *Betaproteobacteria* dominated in Bioreactor and Control 1, accounting for about 54.74% and 33.28%, respectively.

The key microorganisms at the genus level were further investigated (Figure 6c). Compared with inoculum, the relative abundance of *Acidovorax* (23.05%), *Delftia* (19.25%) and *Pseudomonas* (6.41%) were greater in the Bioreactor. *Acidovorax* are iron-oxidizing bacteria, which can destroy the structure of stone coal through iron oxidation and thereby promote metal leaching [43]. Moreover, *Delftia* reportedly exhibited the ability to oxidize vanadium (IV) or vanadium (III) in stone coal with positive oxidase secretion [44]. *Pseudomonas* was reported to be resistant to vanadium and can produce hydrogen cyanide for metal leaching [40,45]. In Control 1, autotrophic sulfur-oxidizing bacteria, *Sulfuritalea*, enriched (2.80%) due to the lack of carbon source. A previous study reported the mechanisms involved in the microbial metal leaching of sulfide minerals. *Sulfuritalea* oxidized metal sulfides to gain electrons directly from minerals [46].

To illustrate the microbial interactions during vanadium bioleaching, a network consisting of the enriched genera was constructed (Figure 6d). The interspecific relationships were predominantly positive, inferring to strong interspecific cooperation within the microbial community to cope with the altered chemical condition during the bioleaching process [47]. *Pseudomonas*, *Delftia* and *Mangroviflexus*, exhibited positive interactions among themselves; however, all of them were negatively associated with *Nakamurella*, which decreased its abundance from 12.19% in inoculum to 0.03% and 0.78% in Bioreactor and Control 1, respectively—possibly because of less metal resistance. *Cellvibrio* and *Terrimonas* were positively associated to *Sulfuritalea*. The latter served as autotrophic bacteria with the ability to fix carbon and synthesize organic acids for the growth of *Cellvibrio* and *Terrimonas*, both of which were heterotrophic bacteria reported to be metal-tolerant and were potential biotic remedial agents for metal contamination [48,49,50].

### 3.6. Evaluation of Practical Implication

The aforementioned results demonstrated the feasibility of employing a mixed-culture consortium to perform the bioleaching of low-grade stone coal. It was noted that the chemical-leaching method usually achieved better performance than bioleaching, with a reported leaching efficiency of V from stone coal up to 76.6% using the deliming-flotation technique [51], 89% recovery using sulfuric acid [52] and 91% recovery with the alkaline leaching process [53]. However, those approaches involved complex processes which were energy intensive and induced secondary pollutions, such as acidic wastewater [17]. An additional assessment was also performed with contemporary studies focusing on bioleaching (Table S3, Supporting Information). In general, bioleaching performances involving heterotrophic bacteria have been rarely recorded. *Sphingomonas desiccabilis* and *Bacillus subtilis* reportedly enhanced vanadium extraction by 184% and 283% compared to the control, respectively, for vanadium bearing extraterrestrial basalt [36]. Heterotrophic bacteria, *Pseudomonas*, and fungi, *A. Niger*, also assisted in vanadium recovery, resulting in 36% and 19% leaching efficiency for pretreated vanadium-rich slag [25]. There were a number of experiments that obtained greater vanadium recovery rates using cultured strains; however, many of which employed acidophile species such as *Acidithiobacillus ferrooxidans* under extreme acid conditions [54,55,56], or targeted material with richer vanadium fractions [39,57,58]. Our study offered an alternative by utilizing indigenous microbiota to support the bioleaching process under circumneutral pH conditions. It may be beneficial to have a mixed culture of microorganisms under heavy metal stress. With greater community diversity, functions that are vital to bioleaching may be well-reinforced by different metabolic pathways as a result of interspecific coordination [54]. In addition, mixed culture is more readily available than pure cultures, mainly utilizing the bacteria living indigenously in the sampled medium, making it more suitable for a practical scenario. However, further effort is necessary to simulate the real-world conditions, such as variation in temperature, pulp density and geochemical conditions. Genera facilitating the bioleaching in the consortium may also require focused study to characterize their specific functions and relevant contribution for vanadium recovery.

## 4. Conclusions

Vanadium could be effectively recovered from low-grade stone coal by aerobic heterotrophic microbial consortium. In the first three cycles, the maximum leaching concentrations of vanadium reached 659 μg/L, 676 μg/L and 707 μg/L, respectively. The recovery of vanadium was increased by 7.24 times as a result of microbial activity. The SEM analysis revealed traces of bacterial erosion with fluffy structures on the surface of the stone coals after the leaching process. XPS analysis showed that the vanadium in the stone coal mainly existed in the form of V(IV) and V(V). Microorganisms undermined the structure of the stone coal and released vanadium. The microbial community analysis found that the abundance of *Acidovorax*, *Delftia* and *Pseudomonas* increased significantly during the leaching process, which reportedly contributed to bioleaching from metal ores. Our work laid a solid ground for the development of mixed-culture-based technology to recover vanadium from low-grade stone coal. Future research is needed in order to test the practicality of scaling up the process for real application.

## Data Availability

The data presented in this study are available on request from the corresponding author. The data are not publicly available due to privacy.

## Supplementary Materials

The following supporting information can be downloaded at: https://www.mdpi.com/article/10.3390/ijerph192013375/s1.
