# Supplementary material for: Heterotrophic Bioleaching of Vanadium from Low-Grade Stone Coal by Aerobic Microbial Consortium
Source: Int J Environ Res Public Health. 2022 Oct 17;19(20):13375. doi: 10.3390/ijerph192013375 (PMC9603648; doi:10.3390/ijerph192013375)
Supplement: Supplementary file 1 [file ijerph-19-13375-s001.zip › ijerph-1960932-supplementary.pdf]

Supporting Information

Heterotrophic bioleaching of vanadium from low-grade stone coal by aerobic microbial consortium

Han Zhang, Jiaxin Shi*, Cuibai Chen, Meng Yang, Jianping Lu*, Baogang Zhang

School of Water Resources and Environment, MOE Key Laboratory of Groundwater Circulation and Environmental Evolution, China University of Geosciences (Beijing), Beijing 100083, P. R. China

*Corresponding author.

E-mail: jiaxin.shi@foxmail.com (J. Shi), Jianping1590@163.com (J. Lu).

This supporting information contains:

number of pages: 6

number of tables: 3

number of figures: 4

Table S1. Chemical composition of the stone coal determined by X-ray fluorescence analysis.

Element	C	O	Si	Al	Fe	Ca	S	Ti	Mg	K	V
Wt (%)	20.99	28.57	32.56	3.37	2.95	1.36	1.50	0.82	0.74	0.98	0.23

Table S2. Alpha-diversity of bacterial communities in the original and cultivated samples.

**System**	**Chao1 index**	**Ace index**	**Shannon index**
Inoculum	400	384	4.24
Bioreactor	261	273	2.69
Control 1	219	200	2.86

Table S3. Compilation of bioleaching tests with operation conditions and performance results.

Recovery method	Vanadium bearing material	Test apparatus	Treatment cycle	Temperature	Initial pulp density	Initial pH	Vanadium Recovery percentage	Leaching concentration	Functional microbes	Reference
Bioleaching utilizing pure culture of heterotrophic microorganisms	Extraterrestrial basalt<br>V: 175 ppm	Pre-cultured; Experimental container	21 days	20-22℃	NA	7	NA	1.71~3.78 μg/L	*Sphingomonas desiccabilis*;<br>*Bacillus subtilis*	[36]
Fungi, heterotrophic, autotrophic strain were utilized for bioleaching	LD converter slag (roasted)<br>V: wt 1.97%	Pre-cultured; flask reactors	15 days	30℃	1%	7	19%<br>36%<br>45%	NA	*A.Niger*<br>*Pseudomonas*<br>*A.thiooxidans*	[25]
Bioleaching with pure culture	Vanadium titanium magnetite<br>V: wt 0.18%<br>Steel slag<br>V: wt 10%<br>Clinker<br>V: wt 10%	Pre-cultured; flask bioreactor	22 days	22℃	1-4%	1.5-2.4	12%<br>23%<br>55%	NA	*Acidithiobacillus ferrooxidans*	[54]
Bioleaching with pure culture	vanadium bearing shale<br>V: wt 0.73%	Pre-cultured; flask bioreactor	10 days	30℃	2%	1.5	45%	NA	*Acidithiobacillus ferrooxidans*	[24]
Bioleaching using mixed acidophilic culture	Steel slag<br>V: wt 0.04%	Pre-cultured; batch reactor	6 days	28 ℃	1%	1	NA	700 μg/L	*A. thiooxidans*<br>*A. ferrooxidans*	[55]
	Steel slag<br>V: wt 0.04%	Pre-cultured; Column reactor	22 days	28 ℃	10%	1	8%	860 μg/L		

Recovery method	Vanadium bearing material	Test apparatus	Treatment cycle	Temperature	Initial pulp density	Initial pH	Vanadium Recovery percentage	Leaching concentration	Functional microbes	Reference
Bioleaching using chemolithotrophic oxidizing bacteria	Vanadium rich spent refinery catalysts 1.4%	Pre-cultured; flask reactor	7 days	32 ℃	0.5%	2	18%	NA	*At.thiooxidansn*	[56]
Bioleaching utilizing pure culture	Vanadium bearing stone coal V: wt 1.39%	Pre-cultured; flask reactors	28 days	30℃	2%	7	60.2%	NA	*Bacillus mucilaginosus*	[57]
Bioleaching utilizing acidophilic bacteria	Vanadium rich spent hydro-processing catalyst V: wt 7.7%	Pre-cultured; batch culture	7 days	32 ℃	5%	1.68	3%	NA	*A.thiooxidans species*	[39]
Bioleaching utilizing gluconic acid yielding genus	Vanadium bearing magnetite V: 3739 ppm	Pre-cultured; flask bioreactor	45 days	20℃	3%	6	3.3%	3059 μg/L	*Gluconobacter oxydans*	[58]

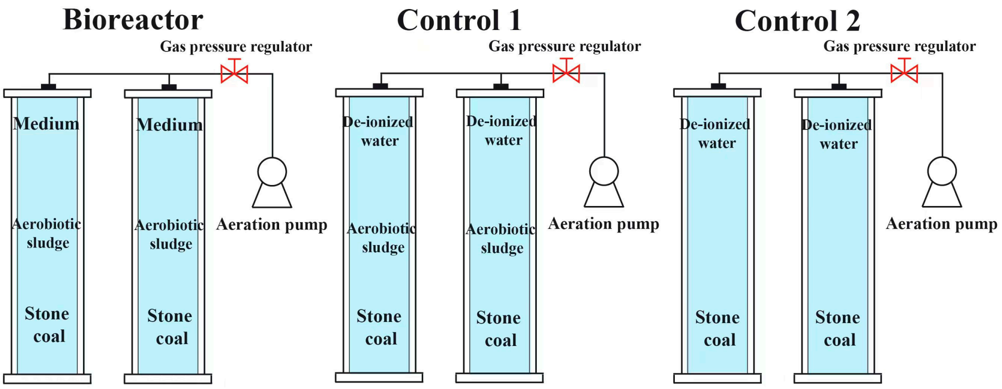

Figure S1. Schematic diagram of bioleaching device.

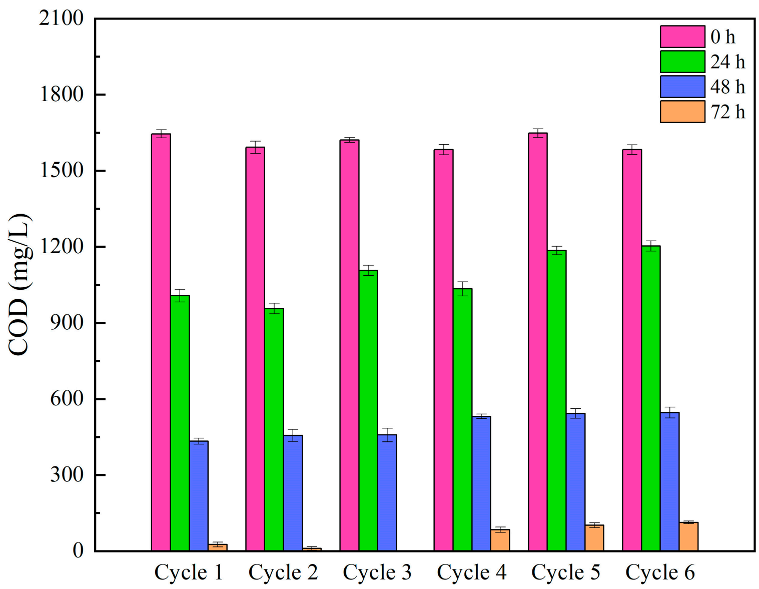

Figure S2. Variations of COD concentration during bioleaching process in Bioreactor.

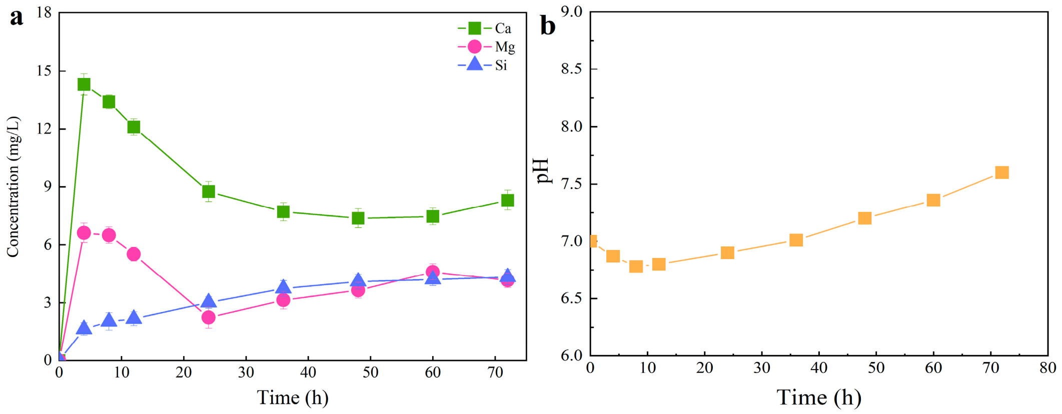

Figure S3. Variations of (a) other main elements concentration and (b) pH level during bioleaching process in Bioreactor.

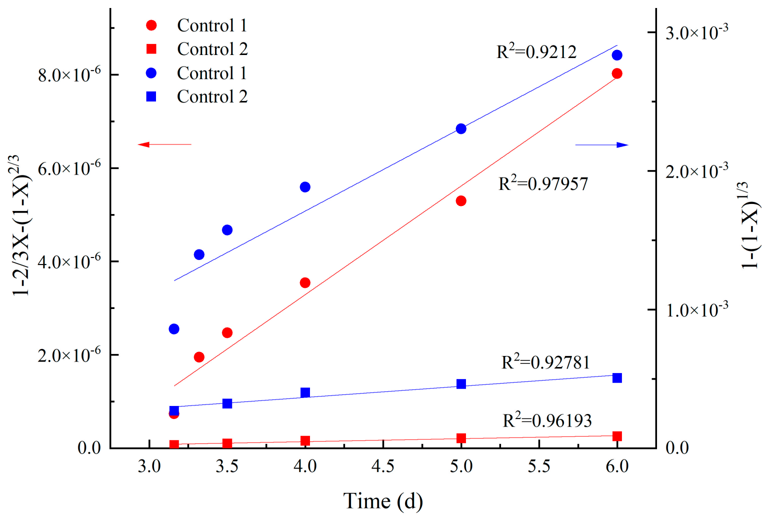

Figure S4. Kinetic model of vanadium leaching controlled by diffusion and chemical reaction in Control 1 and Control 2. left-axis provides measurement label of the diffusion model (red), right-axis corresponds to the chemical reaction model (blue), as indicated by arrow.
